# Phosphorus-doped silicon nanorod anodes for high power lithium-ion batteries

**DOI:** 10.3762/bjnano.8.24

**Published:** 2017-01-23

**Authors:** Chao Yan, Qianru Liu, Jianzhi Gao, Zhibo Yang, Deyan He

**Affiliations:** 1 School of Physics and Information Technology, Shaanxi Normal University, Xi’ an 710119, China; 2 School of Physical Science and Technology, Lanzhou University, Lanzhou 730000, China

**Keywords:** in situ reduction, lithium-ion battery, silicon anode, silicon nanorods

## Abstract

Heavy-phosphorus-doped silicon anodes were fabricated on CuO nanorods for application in high power lithium-ion batteries. Since the conductivity of lithiated CuO is significantly better than that of CuO, after the first discharge, the voltage cut-off window was then set to the range covering only the discharge–charge range of Si. Thus, the CuO core was in situ lithiated and acts merely as the electronic conductor in the following cycles. The Si anode presented herein exhibited a capacity of 990 mAh/g at the rate of 9 A/g after 100 cycles. The anode also presented a stable rate performance even at a current density as high as 20 A/g.

## Introduction

As one of the most popular secondary power sources, lithium-ion batteries (LIBs) are widely used in portable personal electronics, electrical vehicles and grid energy storage because of their high energy and power densities [1–2]. Since the state-of-the-art commercial LIBs are still far from meeting the ever-increasing demands for such applications, LIBs with higher power and energy density are urgently desired [3]. To some extent, the electrochemical performance of LIBs is mainly determined by the two electrodes. The theoretical capacity of commercially used graphite anodes is only 372 mAh/g, which extremely limits the energy density of LIBs [4]. Thus, much attention has been paid to the pursuit of high performance anode materials to replace graphite. Among them, silicon is considered as the most promising alternative due to its high theoretical capacity of 3579 mAh/g (forming Li_3.75_Si at room temperature) and low discharge region (the average delithiation voltage is about 0.4 V) [4–5]. However, unfortunately, such a high capacity is accompanied by huge volume changes, which could directly lead to cracking and pulverization of electrodes during Li-ion insertion and extraction [6]. Numerous results have proven that fabricating nanostructured Si-based anode materials could effectively accommodate the severe volume changes during cycling [7–8]. Lu et al. developed an architecture of flexible silicon and graphene embedded in carbon nanofibers with atomic-scale control of the expansion space as anodes for LIBs. Such an anode delivered an electrochemical performance of 2000 mAh/g at a current density of 700 mA/g [5]. Cui et al. designed a yolk–shell-structured Si anode that has void space between the shell and the particles, allowing for the expansion of Si without deforming the carbon shell. Such an anode shows a capacity retention of 74% after 1000 cycles at a rate of C/10 [9]. Yang et al. fabricated a Si-based anode with a core–shell–shell heterostructure of Si nanoparticles as the core with mesoporous carbon and crystalline TiO_2_ as the double shells. It delivered a high reversible capacity of 1726 mAh/g over 100 cycles [10]. Among various nanostructured Si anodes, the electrodes prepared by depositing Si layers directly on nanostructured current collectors always shows an improved battery performance [7,11]. In such a stratagem, nanostructured current collectors are generally prepared by carbonization of organics, electrochemical deposition with templates, and reduction of metal oxides, all of which are complicated and costly [7,11–13]. On the other hand, as a semiconductor material, the conductivity of Si is not high enough for high-power battery applications. Conductive coatings are usually used to enhance the conductivity of Si-based anodes, but the conductivity inside the Si cannot be changed by this approach, resulting in limited improvements of the high-power performance for Si-based anodes.

In this work, we prepared heavy-phosphorus-doped silicon anodes on CuO nanorods for high power LIBs. In our experiments, once the voltage cut-off window was set in the range that only covers the discharge–charge range of Si, the conductivity of CuO nanorods was highly increased by the irreversible reaction with Li-ion during the first discharge, and the lithiated CuO played the role of an electronic conductor in the following cycles. In addition, the silicon layer was heavily doped by phosphorus and delivered a conductivity as high as 54.0 S/cm, which is vital for the enhanced high power performance of the obtained Si anode.

## Results and Discussion

**Figure 1:**
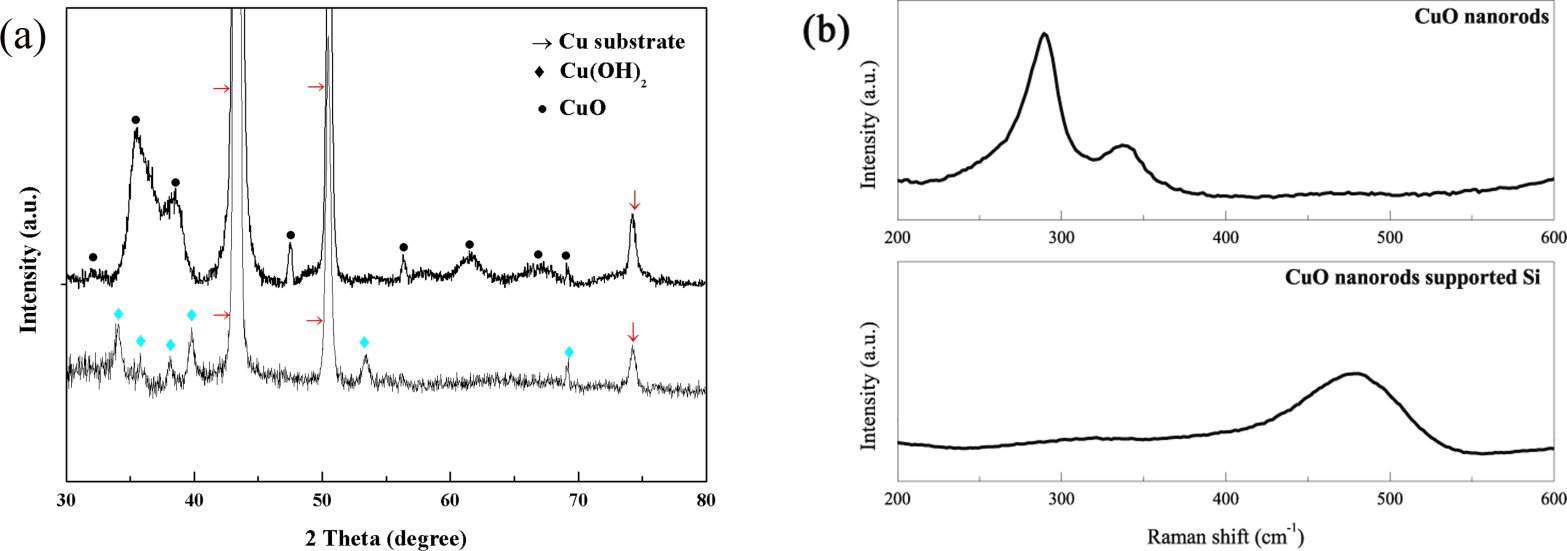
(a) X-ray diffraction patterns of the as-prepared Cu(OH)_2_ and CuO nanorods; (b) Raman spectra of the CuO nanorods and Si-coated CuO nanorods.

The X-ray diffraction patterns shown in Figure 1a proved that the product was Cu(OH)_2_ (JCPDS 13-0420) after electrochemical etching of Cu. The thermal treatment transformed the Cu(OH)_2_ completely into CuO (JCPDS 05-0661). From Figure 1b, it can be seen that the Si layer was totally amorphous.

**Figure 2:**
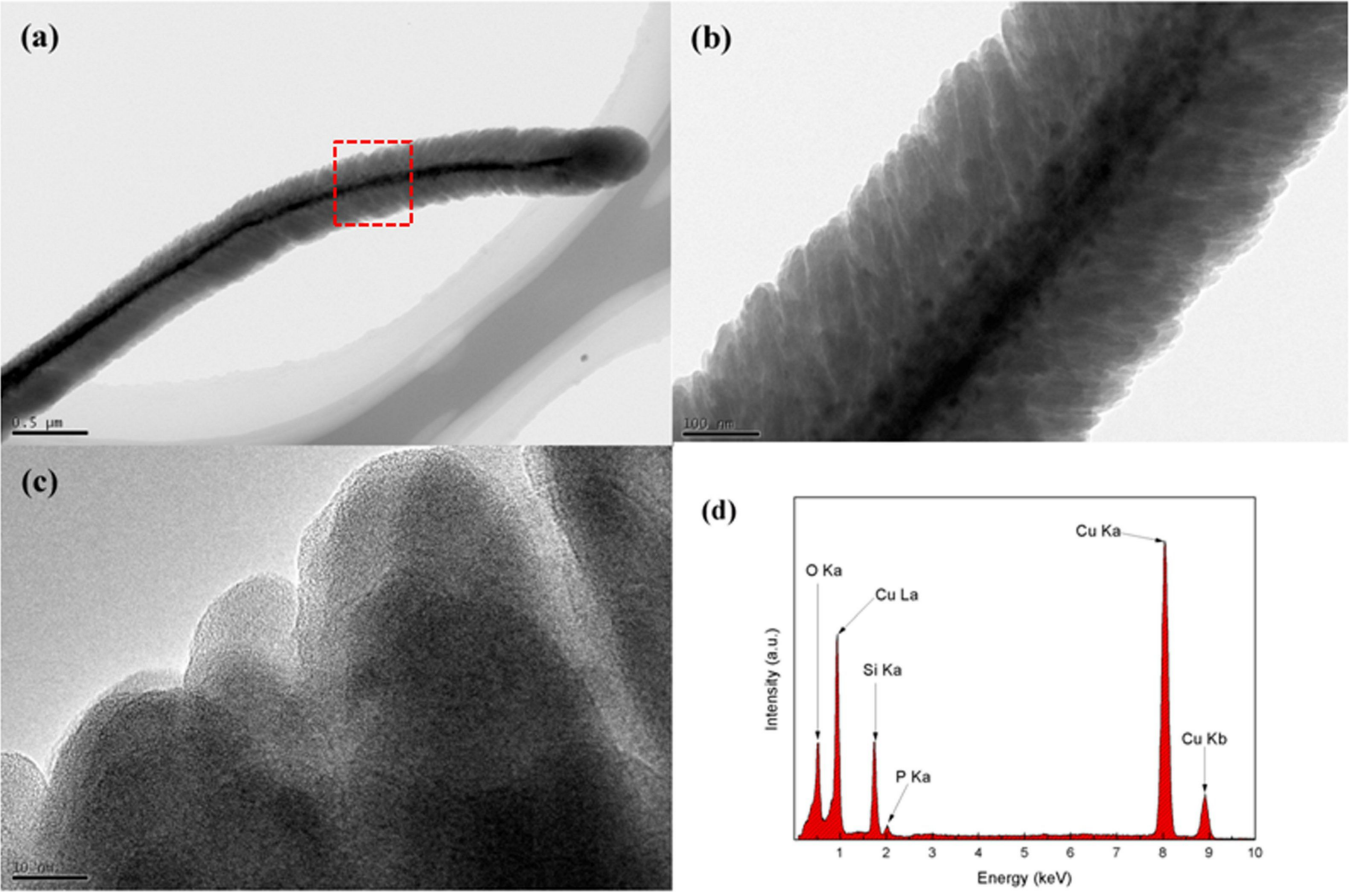
Transmission electron microscopy images for the Si nanorod at (a) low magnification (b) high magnification and (c) high-resolution TEM. (d) Energy dispersive spectra of the Si nanorod section as indicated by the red box in (a).

The structural information of the obtained Si anode was further identified by transmission electron microscopy (TEM). Figure 2a clearly shows that the Si was conformally coated on the CuO nanorods, resulting in a core–shell nanorod structure. From Figure 2b, it can be seen that the thickness of the Si layer was about 150 nm, and the Si shell was composed of small Si columns with significant free space, which is beneficial to accommodate the large volume changes during Li-ion insertion and extraction. The high-resolution TEM image shown in Figure 2c provides direct evidence for the amorphous nature of the Si layer. Li-ions diffuse much faster in amorphous than in crystalline materials. The degradation of the Si anode caused by Li-ion insertion and extraction could also be suppressed in amorphous structures since the volume changes in such a material are more homogeneous [14]. From the energy dispersive (EDX) spectra illustrated in Figure 2d (collected in the region indicated by the red box in Figure 2a), it can be concluded that phosphorus atoms exist in the Si layer with an atomic ratio of about 3%. The existence of phosphorus in the Si layer was further proved by X-ray photoelectron spectroscopy (XPS) analysis as shown in Supporting Information File 1, Figure S1. The two peaks at 130.47 eV and 129.74 eV as shown in Supporting Information File 1, Figure S1 could be ascribed to P 2p_1/2_ and P 2p_3/2_, respectively. Phosphorus-doped Si anodes are assumed to undergo less volume changes than undoped anodes during Li-ion insertion and extraction. This is because some positions are taken by phosphorus and phosphorus is inactive for Li-ion, which means less lithium will be intercalated. Meanwhile, phosphorus doping could highly enhance the conductivity of the Si anode, which is essential for high power performance of LIBs.

**Figure 3:**
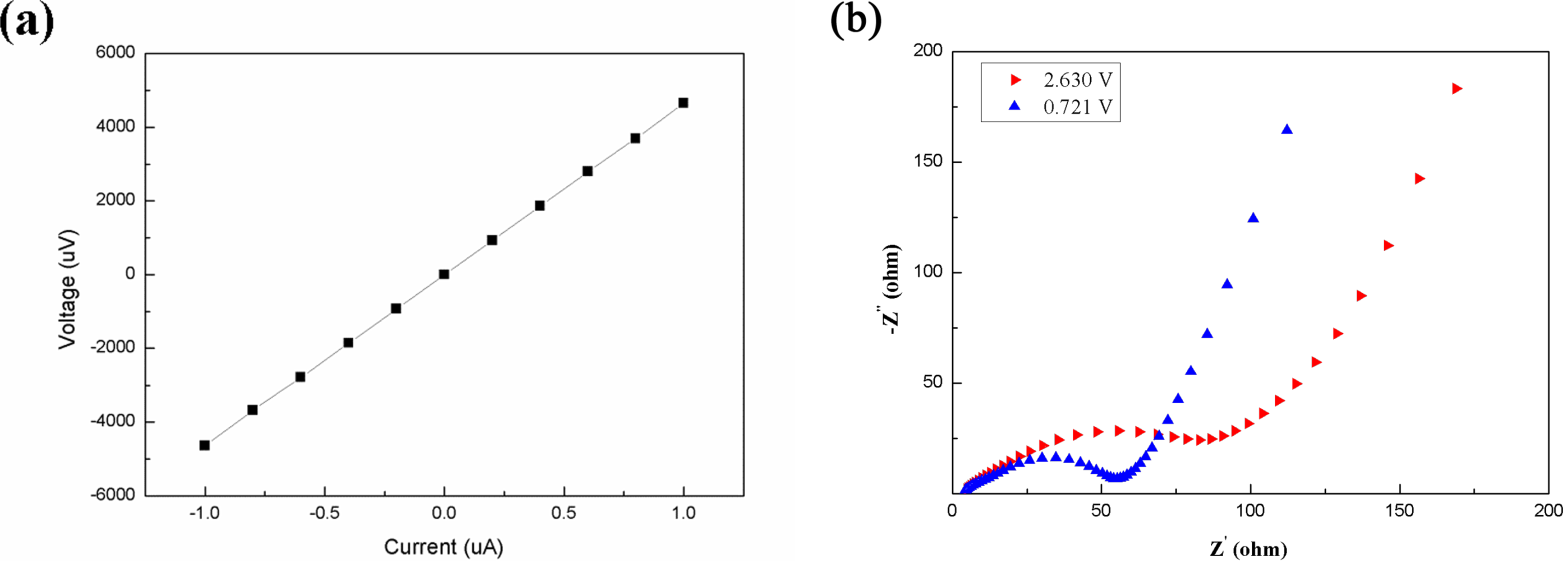
(a) *I*–*V* curve of the as-prepared Si film on glass substrate. (b) Nyquist plots of the Si electrode at 2.630 V and 0.721 V.

To evaluate the conductivity of the as-prepared phosphorus-doped Si, the conductivity measurement was performed at room temperature on a film deposited on a glass substrate under the same experimental conditions described in the experimental section. Before the measurement, coplanar aluminium electrodes with a length of 1 cm and a spacing of 0.5 mm were deposited on the Si film by electron-beam evaporation combined with a mask. Then, after an annealing treatment, the conductivity of the film was measured by a nanovoltmeter (Keithley, Model 2182A) combined with a DC current source (Keithley, Model 6221). The results in Figure 3a indicate that the conductivity of the as-prepared phosphorus-doped Si film was as high as 54.0 S/cm (1.85 × 10^−2^ Ω cm), which is close to that of a conductor. The electrochemical impedance spectra (EIS) was measured at the voltages of 2.6 V and 0.7 V and the Nyquist plots are shown in Figure 3b. At a voltage of 2.7 V, both CuO and Si were not lithiated and CuO could be totally lithiated at 0.7 V. The reduction in the semicircular nature in the Nyquist plot characteristics shown in Figure 3b indicated that the charge transfer resistance of the obtained Si anode at 0.7 V was much lower than that at 2.6 V. According to the equation CuO + 2Li^+^ + 2e^−^ → Cu + Li_2_O, this could be ascribed to the reaction of CuO with lithium, which transformed insulative CuO into conductive Cu. Thus, if the lithiation reaction of CuO is irreversible, the CuO core part could be used as an electronic conductor. For such purpose, the voltage cut-off window was set in the range of 0.02–0.7 V, which avoids the discharge–charge voltage areas of CuO and results in the irreversible lithiation reaction of CuO.

**Figure 4:**
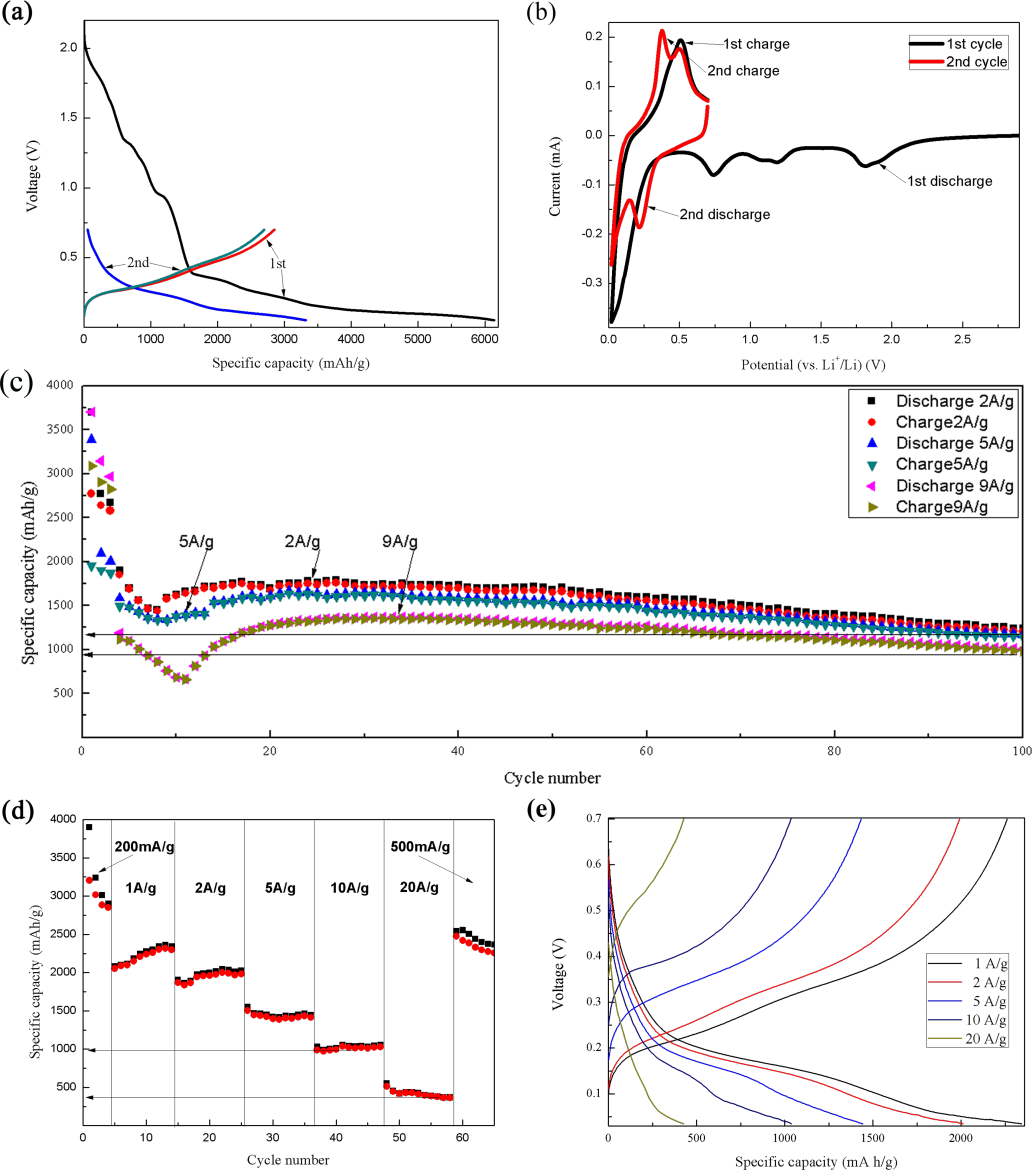
Electrochemical performance of the Si anode. (a,b) Galvanostatic discharge–charge profiles and CV curves for the initial two cycles. (c) Cycling at 2 A/g, 5 A/g and 9 A/g, (d) rate performance, and (e) galvanostatic discharge–charge profiles of Figure 4d.

The galvanostatic discharge–charge profiles given in Figure 4a indicate that the discharge voltage signature of CuO disappears after the first discharge when the voltage cut-off window was set in the range between 0.02–0.7 V, which was further proven by the cyclic voltammetry (CV) curves shown in Figure 4b. From Figure 4a and Figure 4b, it can be concluded that CuO was irreversibly reduced to a conductive mixture of Cu and Li_2_O (according to the equation CuO + 2Li^+^ + 2e^−^ → Cu + Li_2_O) in the first discharge, and by setting the voltage cut-off window in the range only covering the discharge–charge range of Si, the core part of the nanorods could be kept in the conductive mixture state, working only as an electronic conductor in the following cycles. To test the cycling stability of the resulting Si anodes, the anodes were cycled at the current densities of 2 A/g, 5 A/g and 9 A/g (a current density of 200 mA/g was performed in the first three cycles). From the results shown in Figure 4c, it can be seen that the anode delivered a discharge capacity of 1900 mAh/g in the first discharge under the current density of 2 A/g, and such a capacity slowly decreased to 1200 mAh/g after 100 cycles. For the current density of 9 A/g, a reversible capacity of 1100 mAh/g was obtained in the first cycle, which delivered a capacity retention of 90% after 100 cycles. The capacity reduction in the initial 10 cycles can be ascribed to the polarization and peel off of the active Si. After 10 cycles, the structure of the electrodes was stabilized under the test rates, and the activation started to play the leading role in the Si anode. Thus, the increased capacity after the 10th cycle could be ascribed to the activation of the anode. At the same time, it is noteworthy that the enormous specific capacity of Si will bring great changes regarding anode–cathode matching. For the practical application of Si-based anodes, the state-of-the-art commercial cathodes cannot deliver a suitable matching with the Si anode. Thus, the search for suitable cathodes should be of high priority for researchers of Si-based anodes. To match the practical use of Si-based anodes, sulfur (1675 mAh/g) and/or transition-metal-sulphides might be the potential candidates for the state-of-the-art commercial cathodes [15]. In addition, the rate performance of the resulting Si anode was tested and the result is illustrated as Figure 4d. The current density for the test was set at 0.2 A/g to 20 A/g. The prepared Si anode delivered stable cycling performance under all test conditions. It is noteworthy that even under a current density as high as 20 A/g, the Si anode still presented a specific capacity of 380 mAh/g. The galvanostatic discharge/charge profiles of Figure 4d are presented in Figure 4e. It can be seen that the voltage of the discharge region of the Si anode obviously declines as the test rates increase. At the same time, the voltage of the charge region increases. This could be ascribed to the kinetic effects and polarization of the electrode materials, which is a common phenomenon for anode materials of LIBs [16–17].

**Figure 5:**
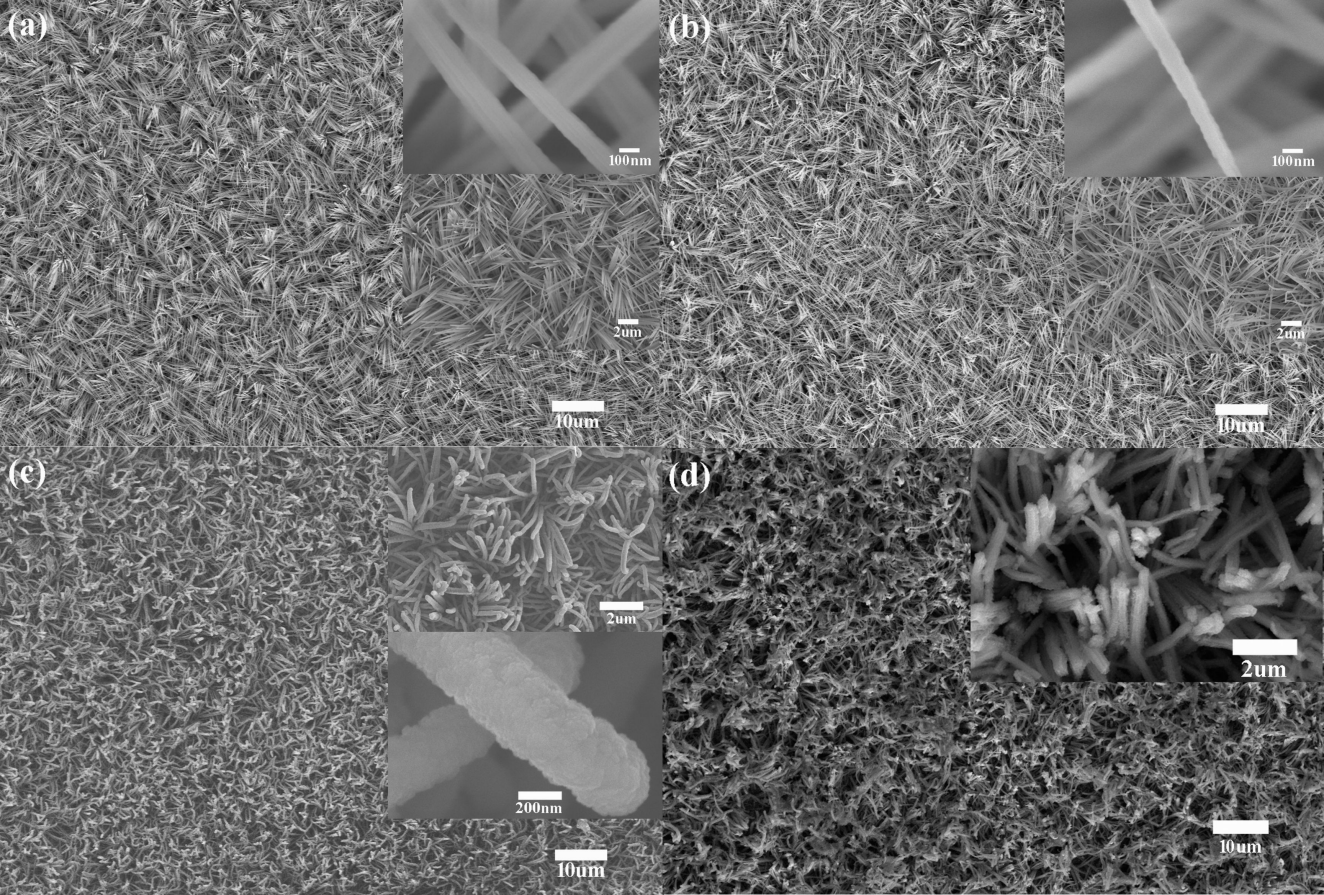
Top view SEM images of the (a) Cu(OH)_2_ nanorods, (b) CuO nanorods, and (c) Si anode supported on CuO nanorods and (d) Si anode after 50 cycles.

The improved lithium storage performance can be ascribed to the unique nanostructure of the as-prepared Si anode. The morphology of the prepared Si anode and the precursors were investigated by SEM. Figure 5a and Figure 5b show the Cu(OH)_2_ and CuO nanorods, respectively. It can be seen that the nanorods are crosslinked and formed a stable three-dimensional network. A comparison between the Cu(OH)_2_ and CuO nanorods indicated that the thermal treatment barely changed the network and the diameter for both of Cu(OH)_2_ and CuO nanorods, which was about 150 nm. The morphology of the obtained Si anode is illustrated in Figure 5c. The Si anode inherited the nanorod structure of the CuO precursor and presented a conformal Si coating. Comparing the insets in Figure 5b and Figure 5c, it can be estimated that the thickness of the Si layer was about 150 nm, which was in accordance with the TEM results. From the cross-section SEM images shown in Supporting Information File 1, Figure S2, the thickness of the active Si nanorods was about 10 µm, which is 29% of the overall thickness of the anode (including the current collector). To verify the structural transformation of the Si anode after cycling, a battery after 50 cycles at a rate of 2 A/g was disassembled. The Si anode was washed thoroughly with deionized water and ethanol to remove the Li_2_O and solid electrolyte interphase layer. The morphology of the cycled Si anode is shown in Figure 5d. Here it is evident that the electrode remained as a nanorod array after 50 cycles without any obvious structural degradation, which may be responsible for the improved lithium storage performance. Based on the above discussion, the improvement of the electrochemical performance of the obtained Si anode could be ascribed to the following factors. First, the transport paths for electrons and Li ions were significantly shortened in the nanorod core–shell-structured electrode. Then, the transport velocity for electrons and Li ions was enhanced by the phosphorus doping for amorphous Si and the Cu conductive core parts of the nanorods, which was directly connected to the substrate. Finally, the free space between the small amorphous Si columns and the nanorods provided enough space to accommodate the volume change caused by Li-ion insertion and extraction.

## Conclusion

In summary, we have successfully fabricated a phosphorus-doped Si anode which delivered improved lithium storage performance. By limiting the voltage cut-off window to the range only covering the discharge–charge range for Si, the CuO core part could be in situ transformed into a conductive mixture of Cu and Li_2_O, which could be used as the electronic collector for the doped Si anode. Such experimental design is promising for simplifying the fabrication procedure of nanostructured current collectors for Si anodes.

## Experimental

### Sample preparation and characterization

The CuO nanorods were obtained by dehydration treatment of Cu(OH)_2_ nanorods at 150 °C in vacuum for 10 min. The Cu(OH)_2_ nanorods were prepared by the galvanostatic electrochemical anodization of a Cu plate in a two-electrode system via a DC power supply (ITECH IT6123B). Typically, a copper foil (20 mm × 20 mm × 25 µm, 99%, back side covered by insulating type) was pretreated in alcohol and then used as the anode. The cathode was a graphite rod with 55 mm length and 5 mm diameter, and was kept 25 mm away from the anode. The anodization process was carried out in 0.8 M NaOH aqueous solution, and a constant current of 6 mA was applied at room temperature. After 12 min, the Cu foil was taken out and washed with deionized water thoroughly. The Si anode was fabricated through deposition of a heavy-phosphorus-doped Si layer directly onto the CuO nanorods via a radio frequency, capacitively coupled, plasma-enhanced chemical vapor deposition (PECVD) device. Typically, the source gas for PECVD was silane (10%, diluted with hydrogen) with a flow rate of 50 sccm mixed with phosphine (5%, diluted with hydrogen) with a flow rate of 5 sccm. The deposition pressure and substrate temperature were 80 Pa and 150 °C, respectively.

The structural and morphological information of the resultant materials were characterized by X-ray diffraction (Rigaku D/Max-2400, Cu Ka radiation), micro-Raman spectrometry (Jobin-Yvon, LabRAM HR800, 532 nm radiation), field emission scanning electron microscopy (FEI, Nova Nano SEM 450), transmission electron microscopy (FEI, Tecnai G2 F30) and X-ray photoelectron spectrometry (VG Scientific, ESCALAB MKII, Mg Ka radiation).

### Electrode preparation and electrochemical characterization

CR 2032 coin type half cells were assembled in an argon-filled glove box (Mikrouna, Super1220, H_2_O and O_2_ <1 ppm) for electrochemical characterization. The obtained Si anode was directly used as the work electrode without any conductive additive and binder. Lithium foil and Celgard 2320 were used as the counter electrode and separator membrane, respectively. The electrolyte was 1 M LiPF_6_ dissolved in ethylene carbonate (EC) and diethyl carbonate (DEC) (1:1 in volume). Galvanostatic cycling was carried out on a multichannel cell test instrument (Neware BTS-610). Cyclic voltammogramms (CV) and electrochemical impedance spectra (EIS) were measured using an electrochemical workstation (Metrohm, Autolab302N). The voltage cut-off window for galvanostatic cycling and CV was set in the range of 0.02–0.7 V. CV tested at a scan rate of 0.1 mV/s. The active mass was evaluated by measuring the mass difference before and after Si deposition through an analytical balance (Mettler, MS105DU, sensitivity of 0.01 mg). The mean loading mass density of Si was about 0.26 mg/cm^2^.

## Supporting Information

File 1 Additional figures.
