# Supplementary material for: Phosphorus-doped silicon nanorod anodes for high power lithium-ion batteries
Source: Beilstein J Nanotechnol. 2017 Jan 23;8:222–8. doi: 10.3762/bjnano.8.24 (PMC5301996; doi:10.3762/bjnano.8.24)
Supplement: File 1 — Additional figures. [file Beilstein_J_Nanotechnol-08-222-s001.pdf]

Supporting Information
for
Phosphorus-doped silicon nanorods anode for high power lithium-ion battery

Chao Yan[‡,1], Qianru Liu[‡,1], Jianzhi Gao[1], Zhibo Yang*[1] and Deyan He[2]

Address: [1]School of Physics and Information Technology, Shaanxi Normal University, Xi’ an 710119, China and [2]School of Physical Science and Technology, Lanzhou University, Lanzhou 730000, China

Email: Zhibo Yang - yangzhb15@snnu.edu.cn

* Corresponding author

‡ These authors contribute equally

Additional figures

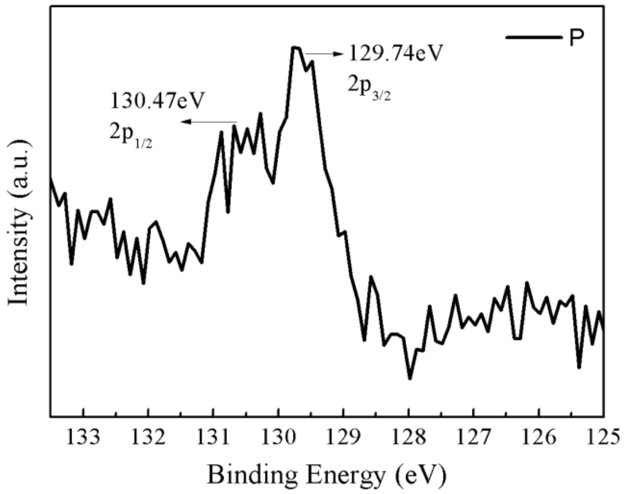

Figure S1: X-ray photoelectron spectroscopy (XPS) of the as prepared Si layer.

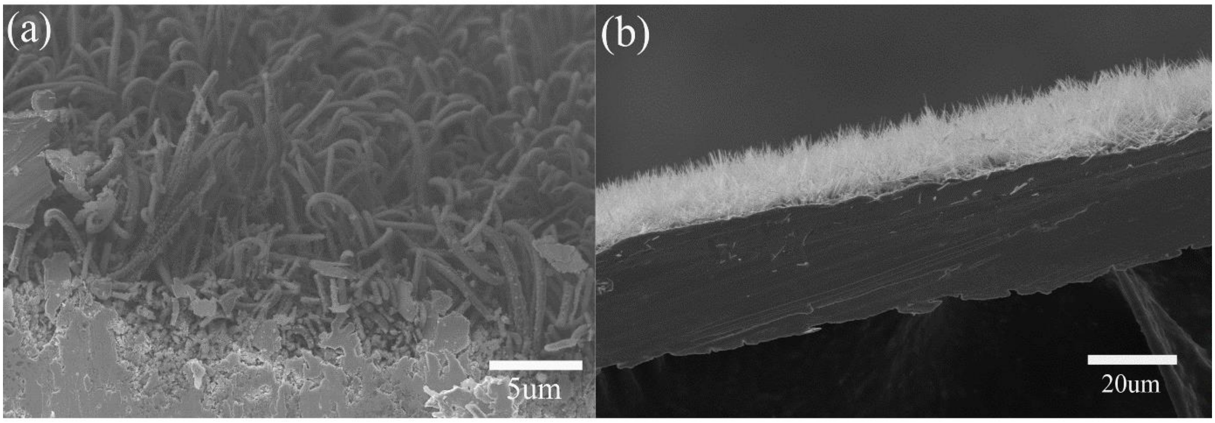

Figure S2: Cross section SEM images for (a) the Si anode and (b) the CuO nanorods.
